# Tibial morphological difference between metal augmentation and actual tibia for revision total knee arthroplasty

**DOI:** 10.1186/s43019-025-00262-9

**Published:** 2025-03-03

**Authors:** Yushi Sakamoto, Shinichiro Nakamura, Yugo Morita, Shinichi Kuriyama, Kohei Nishitani, Sayako Sakai, Yuki Shinya, Shuichi Matsuda

**Affiliations:** Department of Orthopaedic Surgery, Graduate School of Medicine, Kyoto University, 54 Shogoin-Kawaharacho, Sakyo-Ku, Kyoto, 606-8507 Japan

**Keywords:** Total knee arthroplasty, Bone defect, Tibia, Morphology, Metal augmentation

## Abstract

**Background:**

An overhang of the tibial component can cause irritation of the surrounding soft tissues, whereas an underhang is associated with risks of tibial bone resorption. It is not well known whether the tapering angle of currently available blocks at medial, lateral, anterior, and posterior sides matches the actual shape of the proximal tibia. The purpose of this study was to analyze the bony contour of the proximal tibia and measure the tapering angle to examine whether the angle of currently available metal augmentation blocks matches the actual tibia.

**Methods:**

Computed tomography of the lower extremities was performed on 100 consecutive knees, and three-dimensional images of the tibia were reconstructed. The primary resection level was determined on the basis of a plane 10 mm below the center of the lateral tibial plateau. The assumed levels were set to 5, 10, 15, and 20 mm below the primary resection level. All points that were 5, 10, 15, or 20 mm below were projected onto the primary resection surface, and the reduction value from the primary level to each level was measured. The tapering angle was calculated on the basis of the reduction value from the primary level to each resection surface at eight areas and compared with the angle of currently available metal augmentation acquired from the company. The distances of mismatch between the metal augmentation and the bone surface were calculated.

**Results:**

The tapering angle on the medial and lateral sides increased with the more distal resection level, which was up to 30° at the 20 mm level. The tapering angle on the posterior side also increased with the more distal resection level, which was approximately 40° at the 20 mm level. The tapering angle of the current implant was smaller than that of the original tibial morphology. The distances of mismatch varied between implants in which the maximum distance was up to 11.3 mm in the 15 mm augmentation.

**Conclusions:**

The design of current metal augmentation differs from the morphology of the proximal tibia. Surgeons should pay attention to the size mismatch between the femoral and tibial components during revision total knee arthroplasty (TKA).

## Introduction

Treatments for bone defects in revision and primary total knee arthroplasties (TKAs) include modular metal augmentation with a wedge or block, structural allograft, metaphyseal tantalum cone or sleeve, or custom implant [1–3]. Modular metal augmentation is a remarkable option owing to its high availability and familiarity, which restores knee function [4, 5].

Tibial metal augmentation involves a tilt at the medial and lateral sides, as well as the posterior side, especially in a thick block. In the clinical setting, the angle of the augmentation block is different for each implant design. In finite element analyses, metal augmentation is a suitable method for treating tibial bone defects [6]. The morphology of the proximal tibia at the standard resection level has been previously analyzed to provide a suitable tibial component [7, 8]. However, there is limited information regarding the tapering angle of the proximal tibia, and it is not well known whether the angle of currently available blocks matches the actual shape of the proximal tibia. If the tapering angle of tibial augmentation is much steeper than that of the actual bone, the reconstructed surface of the tibia in revision TKA should be smaller than the original surface in primary TKA. The mismatch between the metal augmentation and the bone surface can result in overhang and/or underhang of the tibial component. An overhang of the tibial component can cause irritation of the surrounding soft tissues, leading to reduced postoperative scores [9–11], whereas an underhang is associated with risks of tibial bone resorption, implant subsidence, and loosening [10, 12]. Moreover, the morphology of the proximal tibia can be different between the medial and lateral compartments in which symmetrical metal augmentation does not fit the tibial cut surface at a distal resection level.

Therefore, the purpose of this study was to analyze the bony contour of the proximal tibia at different levels and measure the angle of the taper for each resection surface in the coronal and sagittal planes to suggest the proper tapering angle for tibial metal augmentation. It was hypothesized that the angle of currently available metal augmentation blocks did not match the morphology of the actual tibia and that the angle of the taper could be adjusted on the basis of the thickness and the side of the metal augmentation.

## Materials and methods

### Patient demographics

Consecutive patients who underwent primary TKA for the diagnosis of osteoarthritis were selected. Computed tomography (CT) was routinely performed before surgery for preoperative planning. Patients with a history of surgery on the ipsilateral leg, such as total hip arthroplasty, high tibial osteotomy, or any surgeries for bone fractures, were excluded. Patients with rheumatoid arthritis and severe varus or valgus deformity combined with bone defects during TKA were also excluded. A total of 89 patients (100 knees; 21 men and 79 women) with a mean age of 76.4 years (range, 47–88 years; standard deviation [SD], 6.8) were included in the study. All patients were ethnically Japanese. Their mean height and weight were 154 cm (range, 135–179 cm; SD, 8) and 63 kg (range, 38–94 kg; SD, 12), and their mean body mass index was 26.3 kg/m^2^ (range, 16.3–37.9 kg/m^2^; SD, 4.2). The study was approved by the institutional review board, and informed consent from patients was obtained.

### Measurements

CT of the lower extremity from the hip joint and foot was performed in the supine position with both knees extended, and the images were acquired in 1 mm-thick slices. Three-dimensional images of the tibia were then reconstructed. Using the reconstructed images, the primary resection level was determined on the basis of a plane 10 mm below the center of the lateral tibial plateau perpendicular to the mechanical axis of the tibia. The anteroposterior (AP) axis of the tibia was defined as the line connecting the middle of the posterior cruciate ligament and the medial border of the patellar tendon at the tibial attachment [13, 14]. More distal resection levels were assumed in revision or primary TKA for severe bone defects. In this study, the assumed levels were set at 5, 10, 15, and 20 mm below the primary resection level.

**Fig. 1:**
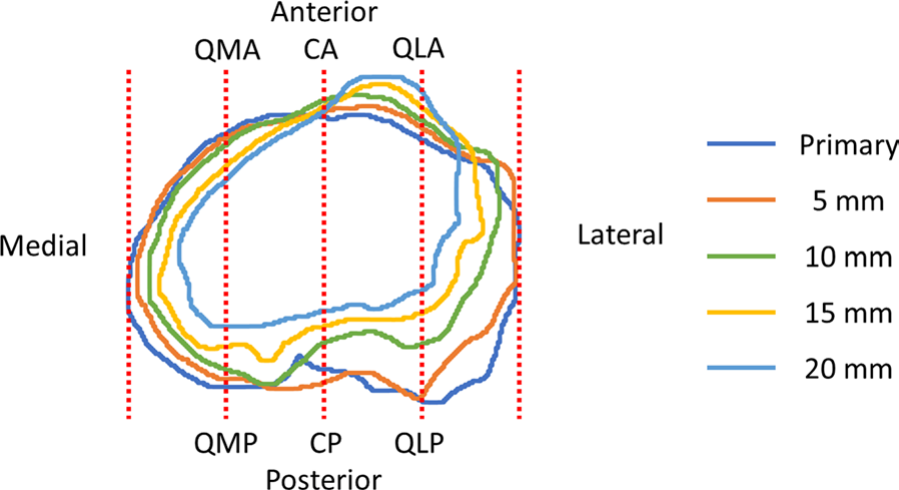
Outlines of the tibial cortex at each level. The six intersection points of the three lines and the tibial contour were named as the QMA, QMP, CA, CP, QLA, and QLP at each resection level. QMA, quarter-medial-anterior point; QMP, quarter-medial-posterior point; CA, central-anterior point; CP, central-posterior point; QLA, quarter-lateral-anterior point; QLP, quarter-lateral-posterior point

**Fig. 2:**
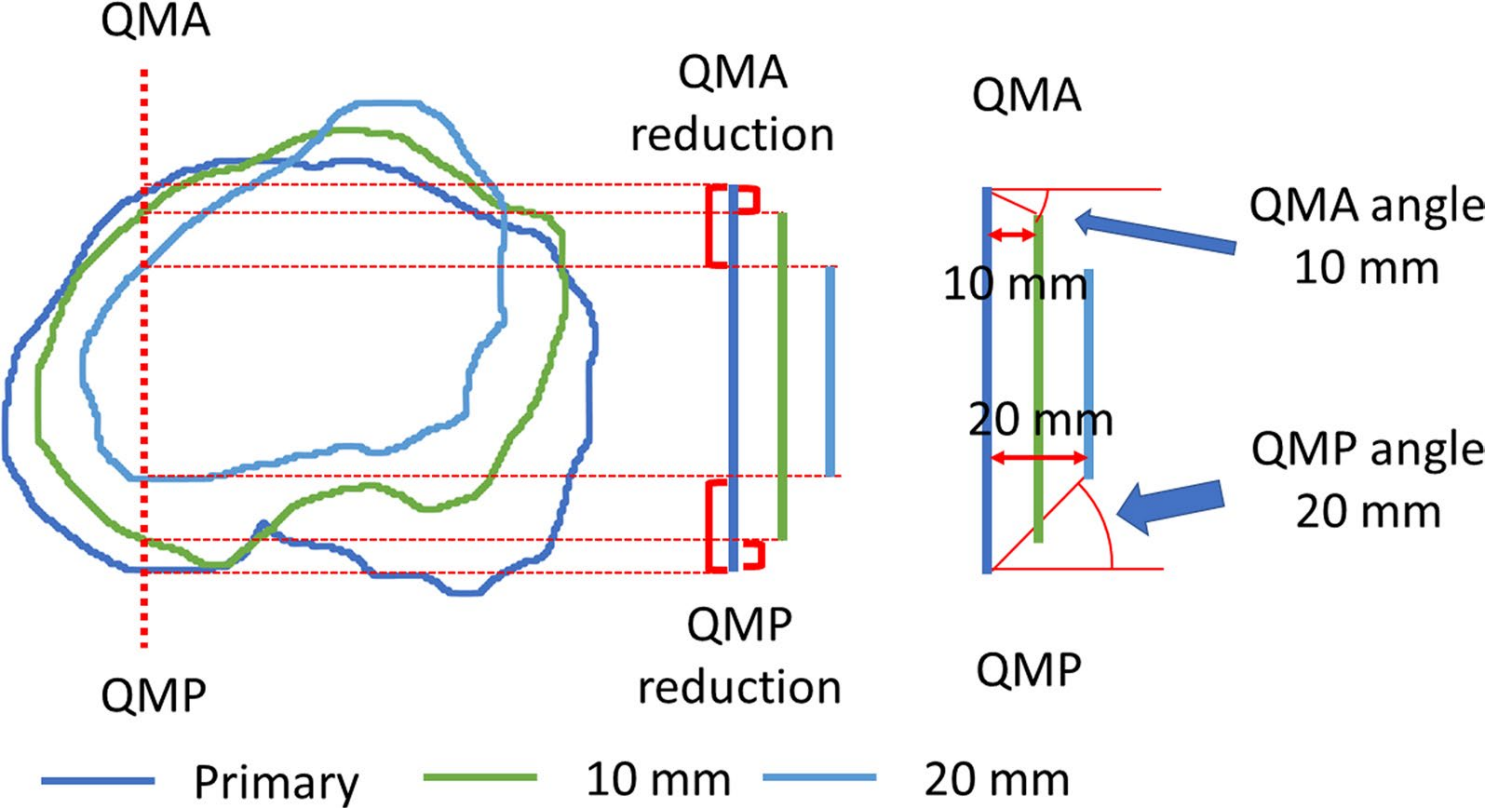
Measurement of the reduction value and tapering angle of the QMA and QMP. Red brackets indicate reduction values at the 10 mm and 20 mm levels. Red arcs indicate the tapering angle at the 10 mm level in QMA and at the 20 mm level in QMP. QMA, quarter-medial-anterior point; QMP, quarter-medial-posterior point

The tibial contour at each resection level was measured using ImageJ software (National Institutes of Health, Bethesda, MD, USA). First, the outline of the tibial cortex was detected at each resection level. During this process, osteophytes surrounding the tibial surface were removed. Second, eight points were determined on the outline of the tibial cortex at each level using the method described below (Fig. 1). The most medial point (Med) and most lateral point (Lat) were defined as the points that were the most distant medially and laterally from the AP axis, respectively. The ML length was defined as the distance between the Med and Lat at the primary resection level. This length was quadrisected, and three lines on the primary cut surface were applied to all resection levels. The six intersection points of the three lines and the tibial contour were named as the quarter-medial-anterior point (QMA), quarter-medial-posterior point (QMP), central-anterior point (CA), central-posterior point (CP), quarter-lateral-anterior point (QLA), and quarter-lateral-posterior point (QLP) at each resection level. Finally, all points that were 5, 10, 15, or 20 mm below were projected onto the primary resection surface, and the distances of the Med, Lat, QMA, QMP, CA, CP, QLA, and QLP from the primary level to 5, 10, 15, or 20 mm below were measured. The distance was defined as the reduction value. The value was denoted as positive if the surface was tapering at the distal level. On the basis of this distance, the angles of the taper were calculated at each of the eight areas from the primary resection surface to each lower resection level (Fig. 2).

The tapering angles of metal augmentation were obtained from the company for three commonly used implants: NexGen LPS-Flex, Vanguard 360, and Persona (Zimmer-Biomet, Warsaw, IN, USA). The tapering angles of the proximal tibial bony morphology were compared with the angle of currently available metal augmentation. The distances of mismatch at the primary resection surface between the metal augmentation and the bone surface were calculated in the ML direction when 5, 10, and 15 mm-thick metal augmentations were used in three different implants.

### Statistical analysis

Intraclass correlation coefficients were calculated to quantify the inter- and intraobserver agreements of the measurements for ten randomly selected knees. The interobserver intraclass correlation coefficients for the Med, Lat, QMA, QMP, CA, CP, QLA, and QLP were 0.814, 0.946, 0.819, 0.910, 0.817, 0.830, 0.860, and 0.917, respectively. The corresponding intraobserver intraclass correlation coefficients were 0.902, 0.959, 0.937, 0.946, 0.811, 0.909, 0.875, and 0.952, respectively. Statistical data were analyzed using two-way repeated analysis of variance to detect differences among each resection level and area. Tukey’s test was applied to the set of all pairwise comparisons. Statistical significance was set at *p* < 0.05. Data analysis was performed using the JMP Pro software version 11 (SAS Institute Inc., Cary, NC, USA).

## Results

### Reduction values

**Fig. 3:**
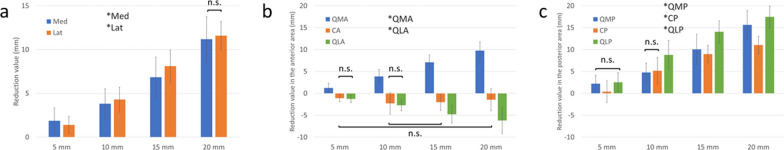
**a** Reduction value of the Med and Lat of the cortex. *Med and *Lat indicate statistical significance at each resection level. Significant differences between the Med and Lat were observed, except at the 20 mm level. n.s., not significant; Med, most medial point; Lat, most lateral point. **b** Reduction value of the QMA, CA, and QLA. The reduction value of the QMA significantly increased with more distal resection, whereas that in the QLA significantly decreased, indicating that the QLA was not tapering. *QMA and *QLA indicate statistical significance at each resection level. n.s., not significant; QMA, quarter-medial-anterior point; CA, central-anterior point; QLA, quarter-lateral-anterior point. **c** Reduction value of the QMP, CP, and QLP. *QMP, *CP, and *QLP indicate statistical significance at each resection level. Significant differences were found between the QMP and QLP, except at the 20 mm level; n.s., not significant; QMP, quarter-medial-posterior point; CP, central-posterior point; QLP, quarter-lateral-posterior point

The average reduction values of the Med and Lat were 1.9 mm and 1.4 mm at the 5 mm level, respectively; 3.8 mm and 4.3 mm at the 10 mm level, respectively; and 6.8 mm and 8.1 mm at the 15 mm level, respectively. Significant differences in the reduction values between the Med and Lat were observed (*p* = 0.010, 0.030, and < 0.001 at the 5 mm, 10 mm, and 15 mm levels, respectively), except at the 20 mm level (*p* = 0.169) (Fig. 3a). On the anterior surface, the reduction value of the QMA significantly increased with more distal resection (*p* < 0.001), whereas that in the QLA significantly decreased (*p* < 0.001), indicating that the QLA was not tapering (Fig. 3b). On the posterior surface, significant differences were found in the reduction value between the QMP and QLP (*p* < 0.001 at the 5 mm, 10 mm, and 15 mm levels), except at the 20 mm level (*p* = 0.192) (Fig. 3c). In most of the medio-lateral, anterior, and posterior bony surfaces, a different contour was observed between the medial and lateral compartments.

### Tapering angles

**Fig. 4:**
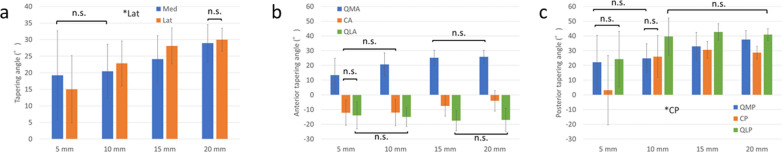
**a** Tapering angle of the Med and Lat of the cortex. Significant differences were observed between the Med and Lat, except at the 20 mm level. *Lat indicates statistical significance at each resection level. n.s., not significant; Med, most medial point; Lat, most lateral point. **b** Tapering angle of the QMA, CA, and QLA. The tapering angle of the QMA was positive at all levels, whereas that of the QLA was negative. n.s., not significant; QMA, quarter-medial-anterior point; CA, central-anterior point; QLA, quarter-lateral-anterior point. **c** Tapering angle of the QMP, CP, and QLP. The tapering angle of the QMP and QLP increased with the more distal resection level in which significant differences were observed between QMP and QLP, except at the 5 mm level. *CP indicates statistical significance at each resection level. n.s., not significant; QMP, quarter-medial-posterior point; CP, central-posterior point; QLP, quarter-lateral-posterior point

The tapering angles of the Med and Lat were also larger at the more distal resection level (Fig. 4a). Significant differences were observed between the tapering angles of the Med and Lat (*p* = 0.016, 0.019, and < 0.001 at the 5 mm, 10 mm, and 15 mm levels, respectively), except at the 20 mm level (*p* = 0.092). The tapering angles of the Med and Lat were approximately 30.0° at the 20 mm level. The tapering angle of the QMA was positive at all levels, whereas that of the QLA was negative (Fig. 4b). The tapering angles of the QMA and QLA at the 20 mm level were 25.8° (SD, 4.6) and −17.0° (SD, 7.9), respectively. The tapering angle of the QMP and QLP increased with the more distal resection level in which significant differences were observed between QMP and QLP (*p* < 0.001, < 0.001, and < 0.001 at the 10 mm, 15 mm, and 20 mm levels, respectively), except at the 5 mm level (*p* = 0.092) (Fig. 4c). The tapering angles of the QMP and QLP were approximately 40.0° at the 20 mm level.

### Morphological comparison with metal augmentations

**Fig. 5:**
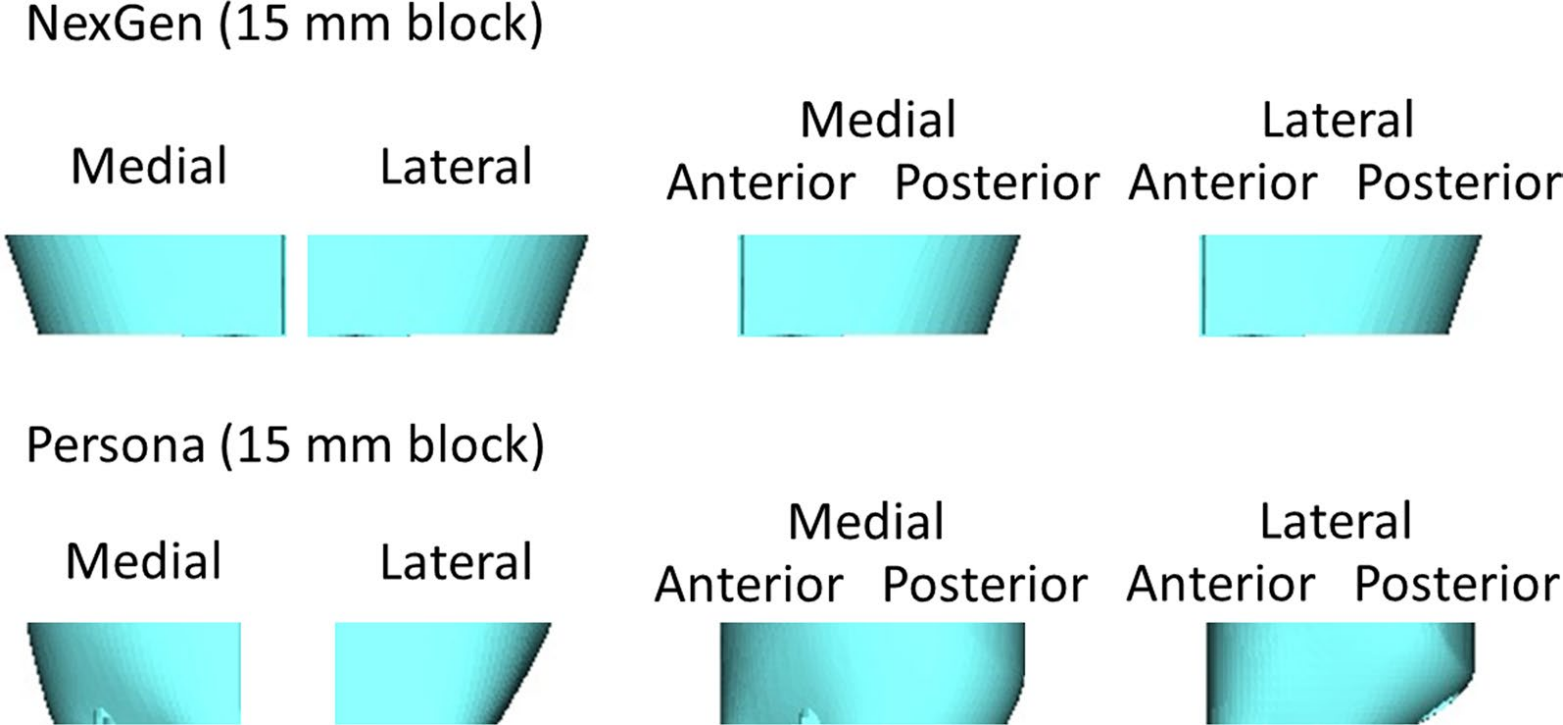
Anteroposterior and lateral views of the 15 mm block in NexGen and Persona

The tapering angles of the conventional implants (NexGen and Vanguard 360) in the 15 mm augmentation were considerably smaller than the tibial bone surface, and there were no tapering angles in the 5 mm and 10 mm augmentations (Table 1, Fig. 5). In Persona, the tapering angle was greater than the conventional implants. However, the tapering angle was still smaller than that of the original tibial morphology. Vanguard 360 and Persona did not have 20 mm augmentations.

**Table 1:** Tapering angle of current metal augmentation and tibia

		5 mm	10 mm	15 mm	20 mm
NexGen	Medial	0°	0°	19°	19°
	Lateral	0°	0°	19°	19°
	Anterior	0°	0°	0°	0°
	Posterior	0°	0°	7°	7°
Vanguard 360	Medial	0°	0°	7°	NA
	Lateral	0°	0°	7°	NA
	Anterior	0°	0°	0°	NA
	Posterior	0°	0°	14°	NA
Persona	Medial	0°	8°	15°	NA
	Lateral	4°	18°	25°	NA
	Anterior	0°	0°	0°	NA
	Postero-medial	0°	0°	8°	NA
	Postero-lateral	0°	18°	29°	NA
Tibia in this study	Medial	19.2° (SD, 13.5)	20.5° (SD, 8.2)	24.1° (SD, 7.1)	28.9° (SD, 5.6)
	Lateral	15.0° (SD, 10.2)	22.9° (SD, 6.7)	28.1° (SD, 5.4)	30.0° (SD, 3.5)
	Antero-medial	13.4° (SD, 11.5)	20.7° (SD, 7.8)	25.2° (SD, 5.1)	25.8° (SD, 4.6)
	Antero-lateral	−13.9° (SD, 9.1)	−15.0° (SD, 6.4)	−17.5° (SD, 6.8)	−17.0° (SD, 7.9)
	Postero-medial	22.1° (SD, 18.3)	24.8° (SD, 9.9)	32.9° (SD, 9.6)	37.6° (SD, 6.1)
	Postero-lateral	24.2° (SD, 18.8)	39.6° (SD, 12.6)	42.7° (SD, 5.6)	40.9° (SD, 4.1)

NA, not available; SD, standard deviation

The distances of mismatch were large in Vanguard 360 for the 10 mm and 15 mm augmentations, reaching up to 11.3 mm in the 15 mm augmentation. In Persona, the distances of mismatch were smallest in these three implants, measuring less than 4 mm in the 5, 10, and 15 mm-thick metal augmentations (Table 2).

**Table 2:** Distances of mismatch between metal augmentation and bone surface

	5 mm	10 mm	15 mm	20 mm
NexGen	3.3 mm	8.1mm	4.6 mm	9.0 mm
Vanguard 360	3.3 mm	8.1 mm	11.3 mm	NA
Persona	2.9 mm	3.5 mm	3.9 mm	NA

*NA* not available

## Discussion

The most important finding of this study was that the tapering angles of the tibia were different between the anterior and posterior areas, medial and lateral areas, and resection levels, which did not match the shape of currently available metal augmentation and varied between implant designs. Therefore, surgeons should pay attention to the size mismatch between the femoral and tibial components during revision TKA to avoid over- or underhang. This study can provide valuable knowledge for designing proper tibial metal augmentation.

Modular metal augmentation with a stem extension is one of the best options among methods for compensating bone defects [1–3, 15, 16]. Metal augmentation is suitable because the stresses in the proximal cancellous bone are low in the finite element analysis [6]. Herein, the bony contour of the proximal tibia was analyzed at different levels, and the reduction values and tapering angles for each resection surface were measured to determine the proper morphology for tibial metal augmentation. An overhang is known to cause irritation of the surrounding soft tissues [9–11]. Overhang of the tibial component on the medial side decreases the Knee Society Score (KSS) and Western Ontario and McMaster Universities Osteoarthritis (WOMAC) pain score 2 years later [10]. In addition, overhang in the posterolateral region negatively affects the KSS and WOMAC pain score [11]. Tibial underhang after surgery is positively correlated with tibial bone resorption 2 years later on the medial and lateral side [10, 12]. Surgeons are recommended to fit a suitably sized tibial component perfectly to the edge of the tibia [12].

In the current study, the reduction values of the Med and Lat were 1.9 mm and 1.4 mm at the 5 mm level, respectively; 3.8 mm and 4.3 mm at the 10 mm level, respectively; and 6.8 mm and 8.1 mm at the 15 mm level, respectively. In the augmentation of 5 mm, no tapering angle can be accepted because the reduction of the ML length was 3.2 mm at the 5 mm level. At the 10 mm level, the reduction of the ML length was 8.1 mm. In NexGen and Vanguard 360, there were no tapering angles in the 10 mm augmentations in which two sizes can be different from the primary surface. The compatibility of the femoral and tibial components can be an issue. At the 15 mm level, the reduction of the ML length was 14.9 mm, whereas the reduction of the 15 mm augmentation in the ML length was 10.3 mm and 3.7 mm in NexGen and Vanguard 360, respectively. The distances of mismatch were 11.3 mm and 4.6 mm in NexGen and Vanguard 360, respectively. In Vanguard 360, the compatibility can still be an issue because three sizes can be different from the primary surface.

Meanwhile, Persona is a relatively new type of implant with an asymmetrical anatomical design. For the 10 mm and 15 mm blocks of Persona, the reduction in the ML length was 4.6 mm and 11.0 mm, respectively. The distances of mismatch were less than 4 mm in all thick metal augmentations. The recent design of metal augmentation can represent the tibial morphology more closely than that of conventional implants (Table 1). However, the tilt was still smaller than that of the original tibial morphology. Surgeons should consider the size mismatch between the femoral and tibial components during revision TKA on the basis of the ML length, and the compatibility of the femoral and tibial components should be checked preoperatively. Moreover, personalized implants can be an option to avoid the size mismatch.

Concerning the design of the anterior and posterior surfaces, most of the designs of metal augmentation for the symmetrical tibial component were symmetrical. However, with a more distal resection level, the ML and AP lengths decreased significantly, and the surface rotated internally [17]. In the current study, the reduction values and tapering angles for the anterior and posterior surfaces differed between the medial and lateral compartments. In comparison with the original surface, the anterior edge of the medial compartment was reduced, whereas that of the lateral compartment protruded owing to Gerdy’s tubercle. The reduction values and tapering angles at the posterior edge were greater in the lateral compartment than in the medial compartment. Asymmetrical metal augmentation would fit the bony surface better and more closely at the distal resection level.

Our study has several limitations. First, the patients included were ethnically Japanese and female-dominant, with relatively short heights and low weights [18–20]. The findings of the study may not be applicable to male patients or patients of other ethnicities, because differences in knee morphology are identified among ethnic groups [21]. To evaluate large tibial baseplates requiring larger metal augmentations, more male participants are required. Second, only patients who underwent primary TKA were analyzed in the study. The morphology may be different in patients with severe bone loss who have undergone revision TKA. Third, the primary resection level was determined 10 mm below the lateral tibial plateau regardless of the patient’s height and fibular level, which was slightly lower than that in other studies [7, 8, 22, 23]. Revision TKA is a very complex operation, and therefore, the level of the tibial plate can be changed depending on the case. If the tibial plate is placed in a lower position and the smaller metal augmentation is used, the distances of mismatch can be smaller. Finally, a review of the literature shows that there is a lack of biomechanical research on tibial morphology, such as tibial overhang and underhang, and that the effect of metal augmentation on biomechanical characteristics has not been investigated. Further biomechanical research should be conducted to investigate potential impacts on stress distribution and implant longevity.

## Conclusions

The design of current metal augmentations differs from the morphology of the proximal tibia. Surgeons should pay attention to the size mismatch between the femoral and tibial components during revision TKA because the size of the tibial component fitted to the more distal level can be smaller with the current metal augmentation.

## Data Availability

The manuscript has no associated data.
